# Clinical and radiological results of treating lumbar spondylosis with cortical bone trajectory screws

**DOI:** 10.1097/MD.0000000000027670

**Published:** 2021-11-05

**Authors:** Mateusz Bielecki, Przemysław Kunert, Artur Balasa, Sławomir Kujawski, Andrzej Marchel

**Affiliations:** a Department of Neurosurgery, Medical University of Warsaw, Warsaw, Poland; b Department of Exercise Physiology and Functional Anatomy, Collegium, Medicum in Bydgoszcz, Nicolaus Copernicus University in Toruń, M. Sklodowskiej-Curie 9, Bydgoszcz, Poland.

**Keywords:** complication, cortical bone trajectory screws technique, mid-term follow-up, minimally invasive spine surgery, traditional trajectory

## Abstract

The cortical bone trajectory screws technique (CBTT) is a popular minimally invasive spine surgery. Few studies have reported long-term outcomes. We aimed to evaluate the complication profile and long-term follow-up results of patients with lumbar degenerative disease treated with the CBTT.

This retrospective analysis included the first 40 consecutive patients that underwent the CBTT. The indication for surgery was critical stenosis of the intervertebral foramen, which required removal of the entire intervertebral joint, on at least 1 side, during decompression.

The last follow-up showed minimal clinically important differences in the numerical rating scale of leg pain, the numerical rating scale of back pain, and the Oswestry Disability Index, in 97%, 95%, and 95% of patients, respectively. Thirty-nine patients completed long-term radiological follow-up. Computed tomography demonstrated solid bone union on 47 (92%) operated levels, collapsed union on 2 (4%) levels, nonunion on 1 (2%) level, and 1 (2%) patient was lost to follow-up. Seven patients experienced complications (4 hardware-related). Three patients required 4 revision surgeries.

The CBTT effectively achieved spinal fusion; over 90% of patients achieved clinical improvement at a mean follow-up of 4.4 years (range: 3–5.75 years).

## 1 Introduction

The cortical bone trajectory screws technique (CBTT) is an alternative method of pedicle screw fixation. CBTT can be applied to many paediatric and adult spinal pathologies, including spondylolisthesis, deformities, failed traditional lumbar pedicle screws, adjacent-segment disease, and trauma.^[1–5]^ In traditional trajectory (TT) transpedicular screw fixation, the trajectory in the transverse plane is from lateral to medial, which requires the application of strong muscle retraction. In the CBTT, the trajectory for screw fixation is reversed. Biomechanical tests on cadavers and animals have shown that the risk of screw plowing is lower with CBTT than with TT screws.^[6–8]^ The present study aimed to evaluate the clinical and radiological outcomes and complications in a group of 40 consecutive patients that underwent CBTT fusion, after a mean clinical follow-up of 52.45 months.

## 2 Materials and methods

**Figure 1:**
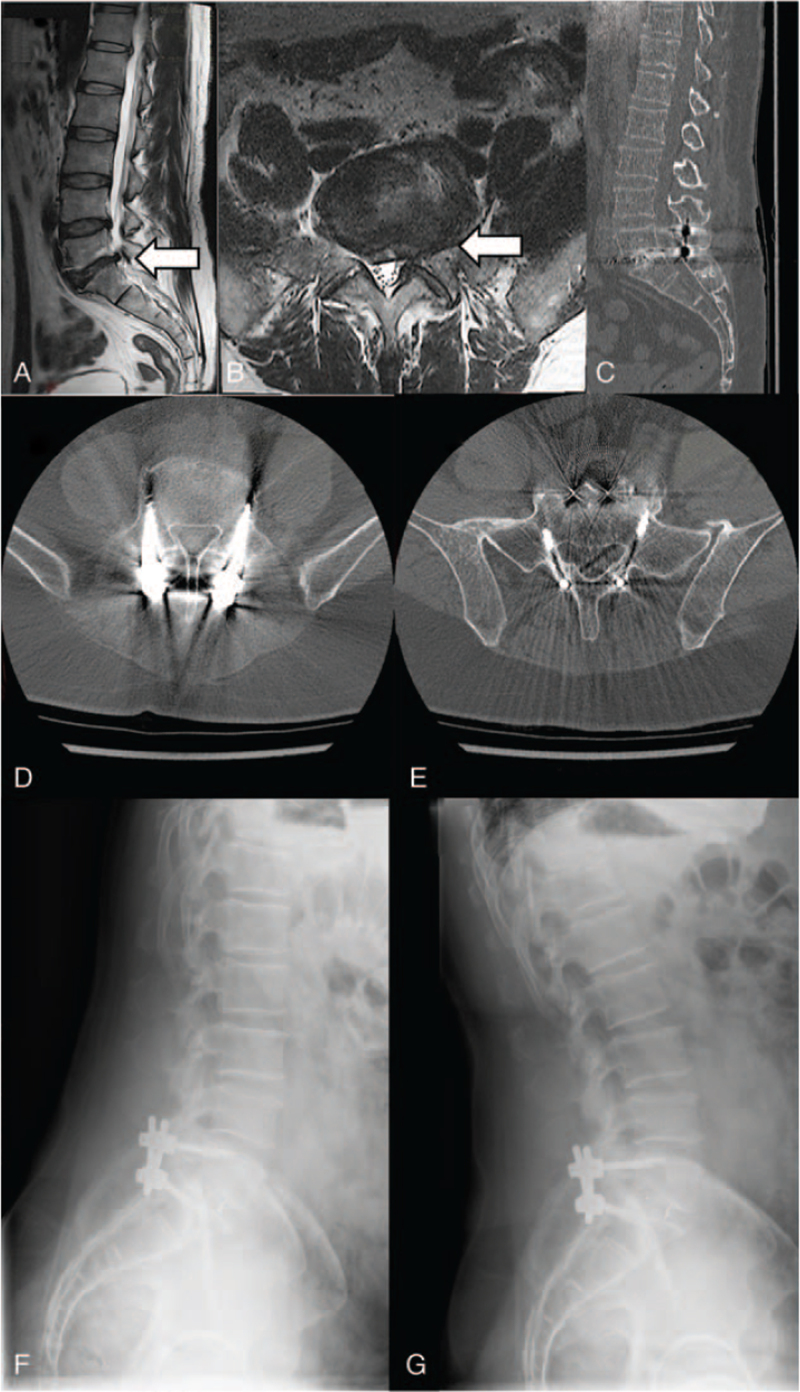
Radiological images of a patient with L5 to S1 central and critical left foraminal stenosis, which presented as left leg radiculopathy. (A) Sagittal T2-weighted magnetic resonance image of the lumbar spine shows a herniated disc at the L5 to S1 level (white arrow). (B) Axial T2-weighted magnetic resonance image shows central and left-sided critical foraminal stenosis at the L5 to S1 level (white arrow). (C) Follow-up sagittal computed tomography scan shows solid fusion status. (D, E) Follow-up axial computed tomography scans demonstrate the correct trajectory of the screws at the (D) L5 and (E) S1 levels. (F), (G) Follow-up lateral dynamic X-rays show no motion at the operated spinal level.

We retrospectively reviewed prospectively collected data on the first 40 consecutive patients that underwent CBTT fusion between 2014 and 2017. Methods of this study were approved by ethics commission of the Medical University of Warsaw on April 4, 2017 under the study approval number: AKBE/159/17. All the patients gave consent to patriciate in this study. Our group comprised 20 (50%) men and 20 (50%) women. The average age of the patients was 60 years (range: 35–86). The symptomatic period varied from 12 to 48 months (mean 23 months). The patient characteristics are given in Table 1. The indication for surgery was critical stenosis of the intervertebral foramen (Fig. 1) that required removal of the entire intervertebral joint, at least on 1 side, during decompression. Among these patients, 13 (33%) had undergone prior microdiscectomies, 16 (40%) had first degree spondylolisthesis, and 10 (25%) had predominant degenerative foraminal stenosis. Treatment results were assessed clinically and radiologically.

**Table 1:** Characteristics of 40 patients with lumbar spondylosis treated with cortical bone trajectory screws.

Characteristic	Category	N (%)
Sex	Male	20 (50%)
	Female	20 (50%)
Age, yr; mean (range)		60 (35–86)
Symptom duration, mo; mean (range)		23 (12–48)
Symptoms	Back pain	37 (93%)
	Sciatica	38 (95%)
	Claudication	25 (63%)
	Paresis	19 (48%)
	Sensory disturbance	25 (63%)
Spondylolisthesis – grade I		16 (40%)
Prior lumbar spine surgery		13 (33%)
Spinal levels of surgery; number of levels (%)	One fusion level	29 (72.5%)
	L4 to L5	23 (57.5%)
	L5 to S1	6 (15%)
	Two fusion levels	11 (27.5%)
	L3–L4–L5	6 (15%)
	L4–L5–S1	5 (12.5%)
	Total number of spinal fusion levels	51
Interbody fusion and CBT details; number of levels (%)	PLIF	28 (55%)
	TLIF	22 (43%)
	Only autogenic graft	1 (2%)
	Total interbody devices, n	78
	Total screws, n	182
Mean operative time, h (range)		3.6 (3–5)

Values are the number of patients (%), unless otherwise indicated. PLIF = posterior lumbar interbody fusion, TLIF = transforaminal lumbar interbody fusion.

### 2.1 Clinical assessment

The average observation time was 52.45 months (range: 36–69). Clinical symptoms were collected at the hospital or through phone calls before surgery and at 3, 12, and 24 months postsurgery, and finally, at the study endpoint, in September 2020. Twelve months after surgery, patients were admitted to the hospital for a short stay to undergo clinical and radiological evaluations. We used the numerical rating scale (NRS) to assess radicular leg pain and back pain. Patient functional status was assessed with the Polish version of the Oswestry Disability Index (ODI) questionnaire.^[9]^ The minimal clinically important difference (MCID) was measured to assess treatment efficiency.^[10]^ The MCID was calculated as the difference between preoperative and different postoperative NRS and ODI values. The MCID was defined as ≥12 points of improvement in the ODI, and ≥3 points of improvement in the NRS.^[11]^

### 2.2 Radiological assessment

Standing radiographs were performed for all patients before discharge to confirm the correct location of the hardware. Long-term radiological control was performed at 12 months after surgery in 39 (98%) patients. Computed tomography (CT) and dynamic flexion-extension X-ray images of the lumbar spine were performed to assess spine stability, mobility of the fused level, bony union, and signs of haloing of the interbody screws. Based on CTs acquired from the sagittal, transverse, and coronal positions, we assessed the positions of interbody screws and the presence of implants, bone union, and screw loosening. Correct screw placement was defined as a cortical screw trajectory that was anchored close (±3 mm) to the disc endplate or the lateral border of the vertebra.^[12]^ Screw position was assessed according to a 2-mm increment grading classification system.^[13]^ Screw loosening was defined as a visible osteolytic lesion on the CT (“halo”) and/or screw migration.^[14]^ Solid bony union in situ was defined as the maintenance of bone continuity between the vertebrae, without signs of graft collapse, on the CT scans.^[15]^ A collapsed union was defined as a solid fusion with ≥2 mm of interbody cage subsidence into an adjacent vertebral body. Nonunion was defined as persistent motion of the fused level on lateral dynamic X-rays. In addition to the scheduled radiological imaging at the follow-up, CTs and magnetic resonance imaging were also performed in patients of significant ongoing pain.

### 2.3 Complications

Complications were defined as early or late. Early complications occurred intraoperatively or during hospitalization. All adverse events that occurred after hospital discharge were defined as late complications. We analysed the data in terms of the following adverse events: screw misplacement, screw haloing, interbody device migration, retroperitoneal haemorrhage, surgical site hematoma, dural tear, infection, new neurological deficit, improper wound healing, thrombosis, adjacent segment disease, and other general complications. Postoperative complications were also analysed according to the Clavien–Dindo classification.^[16]^

### 2.4 Operation technique

**Figure 2:**
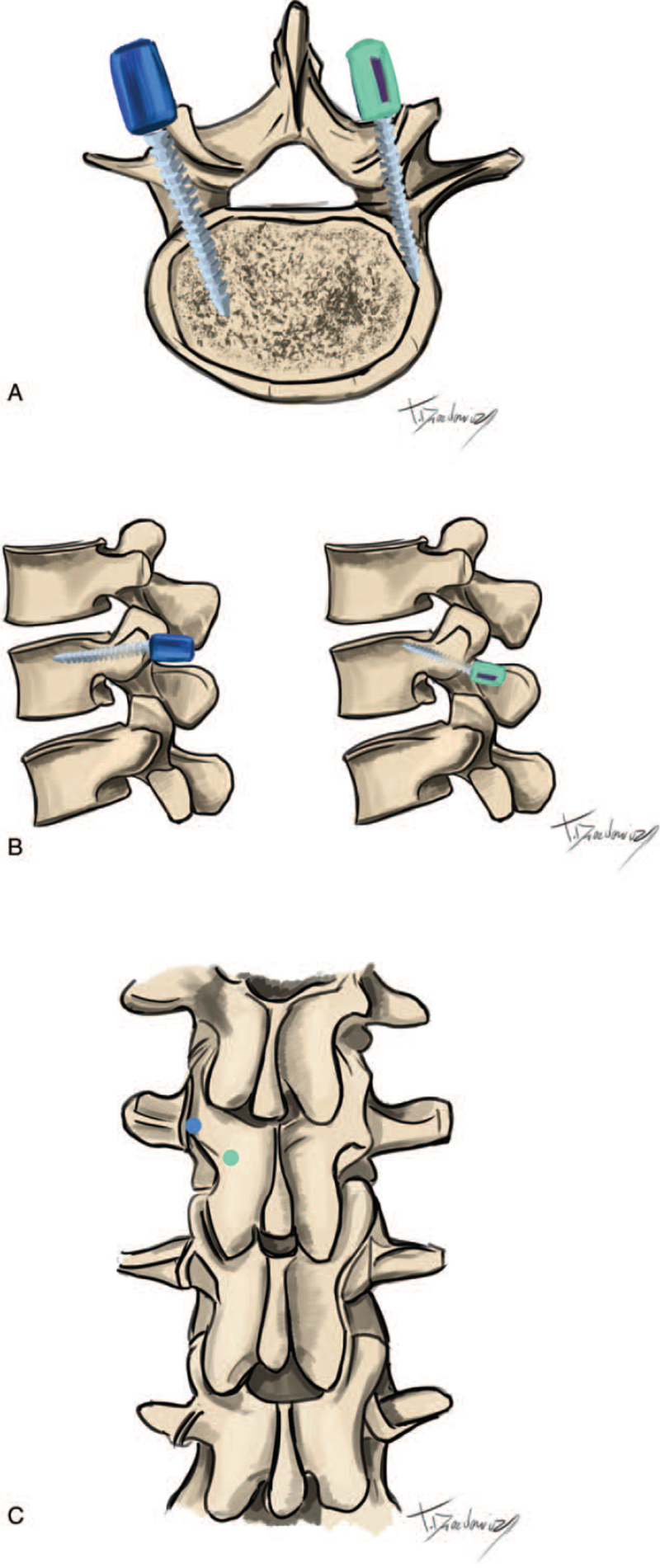
Comparison between screws placed with the cortical bone trajectory and the traditional trajectory. (A) Directions of screws in the traditional trajectory (blue) and the cortical bone trajectory (green) in the axial plane. (B) Directions of screws in the traditional trajectory (blue) and the cortical bone trajectory (green) in the axial sagittal plane. (C) Entry points for screws in the traditional trajectory (blue) and the cortical bone trajectory (green).

With the patient under general anaesthesia and in the prone position, a small incision was made in the midline, above the spinous processes (4–6 cm long). A slight dissection of the spine muscles was performed over the lamina, up to the lateral border of the pars interarticularis, as in a laminectomy, to retain functionality of the neurovascular system in the muscles. Then, 2 anatomical structures were identified: the lateral edge of the intra-articular isthmus, and the lower edge of the transverse process. Fluoroscopy was performed to prepare the starting points for screw insertion at the level of 10 mm. Starting points were located at 1 to 2 mm medial to the connection between the lateral edge of the intra-articular isthmus and the lower edge of the transverse process, directly below the upper connection of the joint surfaces (Fig. 2). Next, the spinal canal was decompressed, and a laminectomy, foraminotomy, and discectomy were performed. Interbody fusion was performed with autogenic local bone chips combined with hydroxyapatite nanoparticle gel (Nanogel, Teknimed, France) and interbody implants. The screw trajectory was controlled with C-arm fluoroscopy. For the S1, we implemented the technique proposed by Matsukawa et al,^[12]^ where the starting point was in the middle of the superior articular process of S1, 3 mm below the inferior articular process of the L5 vertebrae. Then, the screw was drilled along a trajectory that pointed straight along the horizontal plane and at an angle of 10° in the cephalic direction in the sagittal plane. We considered a screw to be well anchored when it perforated the disc endplate or the lateral border of the vertebra, up to 3 mm.^[12]^

### 2.5 Statistical analysis

The Shapiro–Wilk test was used to examine the normality assumption. The Friedman ANOVA test was used to examine the influence of surgery, with the STATISTICA 13.1 program (StatSoft, Inc.). The Durbin–Conover test and the Benjamini & Hochberg *P*-adjustment method were applied for the posthoc analysis. Violin graphs were created with RStudio (version 1.2.5019) and a ggstatsplot library.

## 3 Results

### 3.1 Clinical outcomes

Clinical follow-up was completed in all 40 patients (Table 2). At the most recent follow-up, the average NRS scores for leg pain and back pain decreased by 6 points (range: 1–9) and 5 points (range: 0–10), respectively, compared to the preoperative scores (*P* < .00001 for both). Moreover, the average ODI score decreased by 28 points (range: −2 to 40) (*P* < .00001) compared to the preoperative score.

**Table 2:** Clinical and radiological results of cortical bone trajectory treatment for lumbar spondylosis.

Clinical results	Before surgery	3-month follow-up	12-month follow-up	24-month follow-up (40/40 patients)	Most recent follow-up	Difference between presurgery and most recent follow-up
NRS leg: Range:	7.3 (1–10)	2.9 (0–8)	1.9 (0–7)	1.3 (0–4)	1.3 (0–4)	6 *P* < .00001
NRS back: Range:	6.8 (1–10)	3.2 (0–7)	2.2 (0–7)	2.1 (0–7)	1.8 (0–6)	5 *P* < .00001
ODI: Range:	42 (20–48)	26 (12–40)	17 (0–40)	13 (0–34)	14 (0–34)	28 *P* < .00001
MCID NRS leg		34/38 (89%)	35/38 (92%)	37/38 (97%)	37/38 (97%)	
MCID NRS back		29/37 (78%)	34/37 (92%)	33/37 (89%)	35/37 (95%)	
MCID ODI		30/40 (75%)	36/40 (90%)	37/40 (93%)	38/40 (95%)	

Radiological results	Grade	N/total (%)
The 2-mm increment classification system of screw placement accuracy^[13]^	Grade I	153/182 (84%)
	Grade II	21/182 (12%)
	Grade III	2/182 (1%)
	Grade IV	2/182 (1%)
	LTFU	4/182 (2%)

LTFU = lost to follow-up, MCID = the minimal clinically important difference, NRS = numerical rating scale, ODI = Oswestry Disability Index.

**Figure 3:**
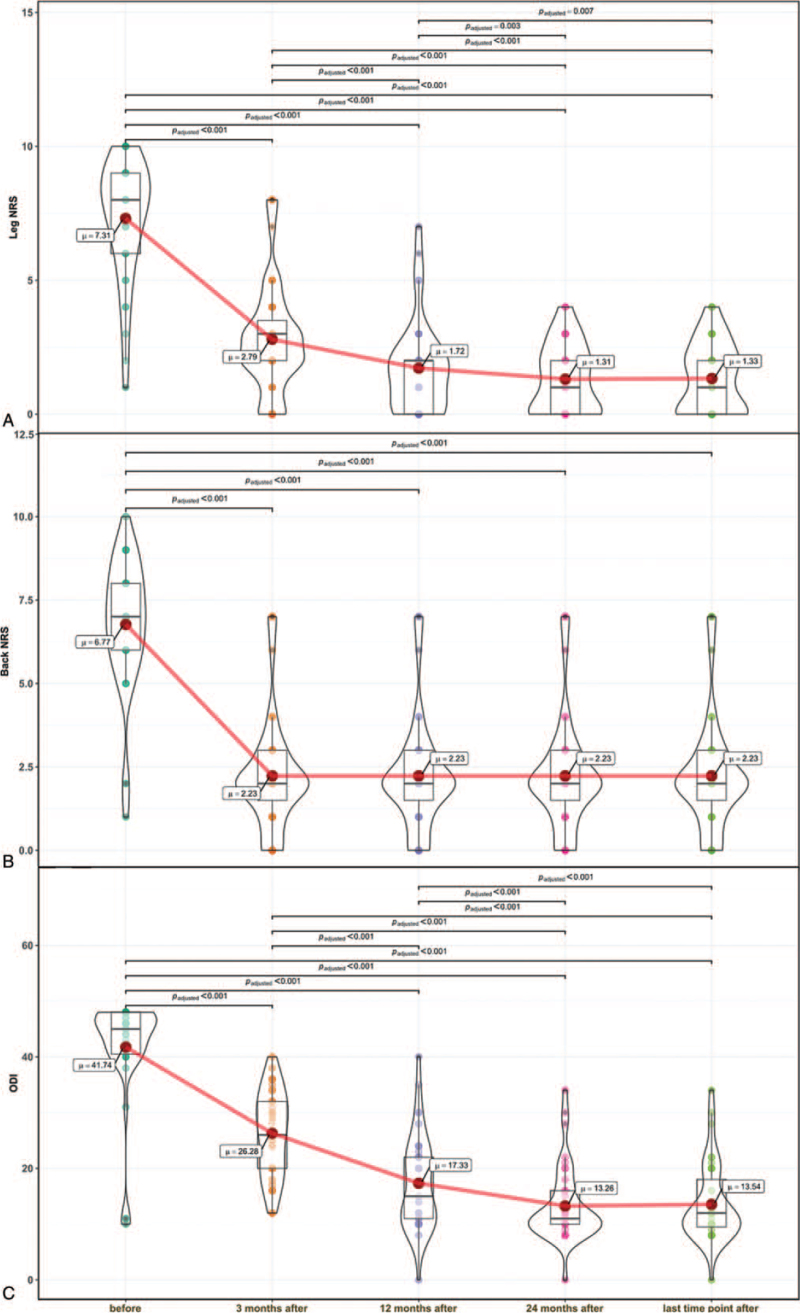
Pain scores measured before and after surgery. Measurements taken before and at different times after surgery show the changes in the average (A) leg numerical rating scale (NRS), (B) back NRS, and (C) Oswestry Disability Index (ODI) score. Red dots connected by the red line indicate the mean value; horizontal black line inside the box denotes the median value. Green dots before and orange dots after surgery denote the results of individual patients. The violin shapes indicate the distributions of results.

A posthoc analysis indicated that, compared to preoperative scores, the average NRS for the leg, NRS for the back, and ODI scores significantly decreased after 3 months, after 12-months, after 24-months, and at the most recent follow-up. Additionally, measurements taken at postoperative intervals showed that the average leg NRS and ODI scores steadily improved over time (Fig. 3A and C). In contrast, the average back pain decreased significantly at 3 months after surgery, compared to presurgery, then remained constant (Fig. 3B). The MCIDs for leg pain NRS, back pain NRS, and the ODI were achieved at the most recent follow-up in 97%, 95%, and 95% of patients, respectively.

### 3.2 Radiological outcomes

**Figure 4:**
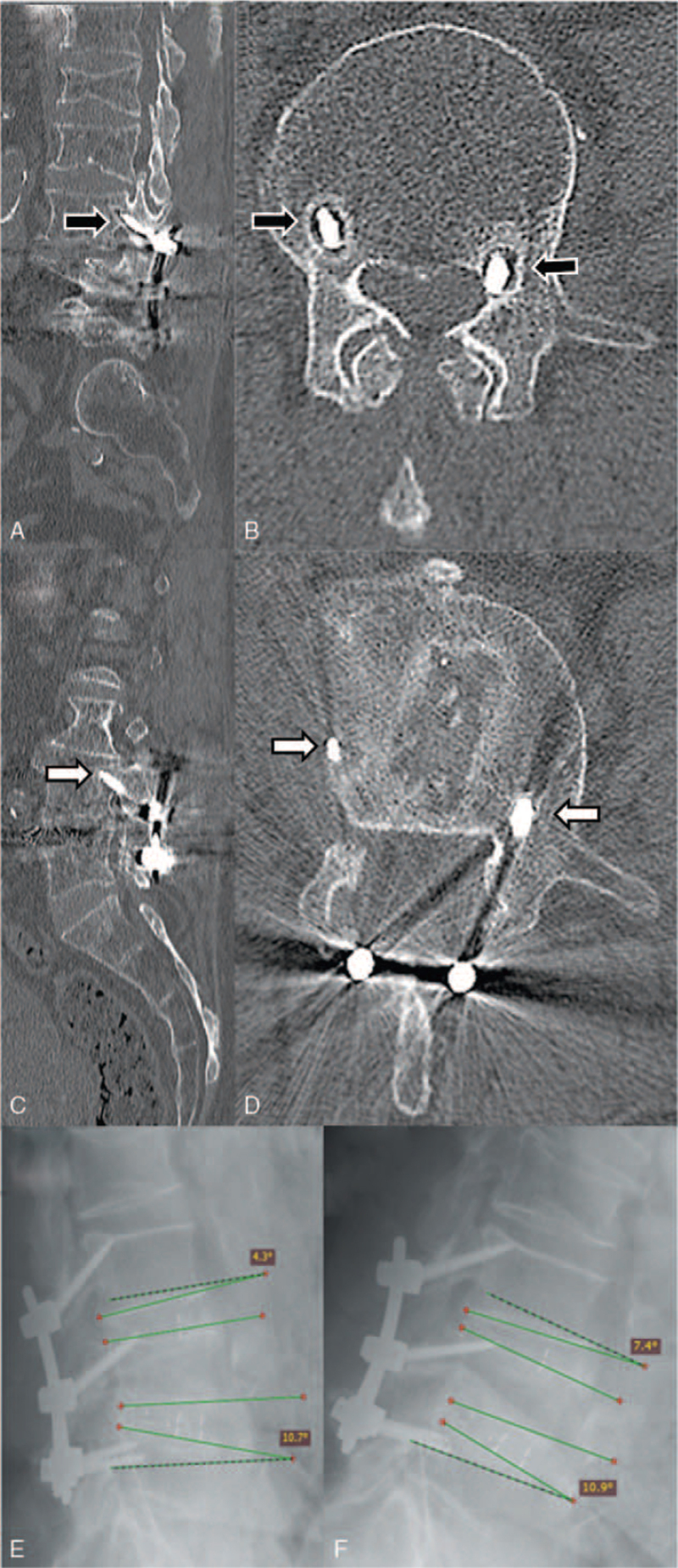
Images of a patient after L3–L4–L5 surgery show representative complications. (A, B) Sagittal and axial computed tomography scans show evidence of haloing around the screws through the L3 pedicles (black arrows). (C, D) Sagittal and axial computed tomography scans show no loosening of the screws through the L4 pedicles (white arrows). (E, F) Sagittal dynamic X-rays in (E) extension and (F) flexion show screw mobility at the L3 to L4 level.

We found no hardware abnormalities in the early postoperative radiographs for our series. However, the follow-up CTs showed that hardware abnormalities occurred in 2 patients. Long-term radiological follow-up imaging was achieved in 39 patients (Table 2). One patient refused follow-up examinations, due to an oncological disease. In total, 50 fused levels, 178 screws, and 77 interbody devices were evaluated in long-term dynamic X-rays and CT scans. Asymptomatic mobility at the fused level was observed in a dynamic X-ray for 1 patient (Fig. 4E and F). Follow-up CT scans showed that solid bone union in situ was achieved at 47 (92%) operated levels, collapsed unions occurred at 2 (4%) levels, a nonunion occurred at 1 level, and 1 patient (1 level, 4 screws) was lost to follow-up. CT imaging also showed that 174 (98%) out of 178 initially placed screws remained in the correct position without signs of “haloing” (Fig. 4C and D). Three (1.7%) screws loosened with signs of “haloing” in 2 patients (Fig. 4A and B). Two (1.1%) screws and 1 (1.3%) interbody device were malpositioned.

### 3.3 Complications

Complications occurred in 7 (17.5%) patients (Table 3). Of these, 3 experienced more than 1 complication. However, only 4 (10%) patients had hardware-related complications. Only 5 patients displayed early complications, including intraoperative dural tears; of those, 3 (60%) patients had previously undergone surgery at the same level. No patient experienced an intraoperative pedicle fracture or damage to the nerve root. Moreover, no other postoperative events occurred.

**Table 3:** Complications of cortical bone trajectory treatment for lumbar spondylosis.

Patient	Early/late	Description	Management
1	Late	1 screw loosened with interbody device dislocation	Reposition of screw in left S1 pedicle and removal of interbody device
2	Early	Dural tear	Suturing + TachoSil
	Late^∗^	Screw malposition	Reposition of screw in left L5 pedicle
	Late	Adjacent segment disease	L3 to L4 laminectomy
3	Early	Dural tear	Suturing + TachoSil
	Late^∗^	Screw malposition	Removal of screw in right L4 pedicle
4	Late	2 screws loosened (asymptomatic)	no treatment needed
5	Early	Dural tear	Suturing + TachoSil
6	Early	Dural tear	Suturing + TachoSil
7	Early	Dural tear	Suturing + TachoSil

Summary of complications				
Totals	Time	Type	N/total (%)	Management
11 adverse events in 7 patients; 178 screws; 77 devices	Early – 5/40 patients	Dural tear	5/40 (12.5%)	Intraoperative repair – 5
	Late – 6/39 patients	Screw loosening	3/178 (1.7%)	4 revision surgeries in 3 patients
		Screw malposition	2/178 (1.1%)	
		Interbody device dislocation	1/77 (1.3%)	
		Adjacent segment disease	1/39 (3%)	
		Other general complication	0 (0%)	

∗ According to our definition, this was a late complication, because the pain presented after hospital discharge.

Late complications occurred in 4 patients. One patient with severe back pain and radicular pain in the left leg displayed screw loosening and interbody device dislocation. Symptoms occurred after physical therapy, 1 year after surgery. This patient required screw replacement in the left S1 pedicle, with a new CBTT procedure, and the interbody device was removed. The second patient experienced severe left leg radicular pain several days after discharge. The magnetic resonance imaging and CT showed foraminal compression of the L5 root, related to an incorrect screw trajectory in the left L5 pedicle, which was not clearly visible in intraoperative and early postoperative X-rays. The patient underwent surgical screw repositioning in the left L5 pedicle. This patient also developed symptomatic central stenosis on the adjacent segment above the fused segment. This complication required an L3 to L4 laminectomy without additional fusion, 17 months later. The third patient experienced severe right-sided pain in the lumbar spine 11 months after surgery. One screw misplacement was found in the right L4 pedicle on the CT of the lumbar spine. The screw was surgically removed on that side without additional fixation, because there were no signs of mobility on that level in a follow-up dynamic X-ray. The fourth patient displayed asymptomatic loosening of 2 screws in a follow-up CT, 12 months after surgery. All of this complications were assigned as type IVb according to the Clavien–Dindo classification.

## 4 Discussion

Cortical bone trajectory has gained popularity as a minimally invasive spine surgery in recent years. The main advantage of CBTT is that foraminal decompression of the spinal canal, discectomy, interbody fusion, and screw fixation can be performed with only 1 small incision. In general, the advantages of minimally invasive spine surgery are less tissue damage, reduced morbidity, faster functional recovery, and the ability to achieve the same surgical goal, compared to traditional surgical methods.^[17]^ Compared to TT screws, CBTT preserves a larger group of muscles, because CBTT does not require the exposure necessary to access traditional screw entry points; instead, only the pars interarticularis must be accessed. However, studies with longer follow-ups are needed for more accurate comparisons. The primary objective of this study was to demonstrate the long-term results of CBTT fixation (mean follow-up 4.4 years). To our knowledge, this study was the first to describe such a long follow-up.

### 4.1 Clinical outcomes

The outcomes, demographic data, and fusion levels of our group were similar to those reported in other studies that had 2 or more years of follow-up.^[2,18,19]^ Chin et al^[18]^ described 30 patients with an average follow-up of 2 years. They reported improvements in the mean visual analogue scale (VAS) for back pain (from 7.8–2.5), in the mean VAS for leg pain (from 4.2–0.2), and in the mean ODI (from 40.8–28.7). In a series of 35 patients, Lee and Ahn^[19]^ showed improvements in the mean VAS for back pain (from 7.7–2.7), the mean VAS for leg pain (from 5.9–1.3), and the mean ODI (from 35.1–11.8).

### 4.2 Radiological outcomes

Solid fusion was achieved in 92% of operated levels; this rate was comparable to those reported in previous studies.^[1,2]^ Sakaura et al^[2]^ reported solid bone fusion in 90.9% of operated levels after single- and two-level fusions in a group of 22 patients. They found nonunions in 4 patients, but none required revision surgery. In contrast, Hussain et al^[20]^ reported a fusion rate of only 37.5% among follow-up CT scans performed at an average 15 months postsurgery. They suggested that their low rate might have been explained by a high rate of undiagnosed osteopenia or osteoporosis, due to the large number of postmenopausal women included in their groups.

### 4.3 Complications

We observed 11 adverse events in 7 patients of our group. However, only 4 (10%) patients experienced hardware-related complications. Dural tears occurred in 5 patients, but 3 of those patients had undergone a previous surgery at the same level. Previous surgery is a clear risk factor for dural sac violations.^[21]^ In the current literature, dural tears have been reported in 4% to 15.6% of patients.^[2,22,23]^ In our group, 2 (5%) patients experienced screw loosening, but one had a diagnosis of osteoporosis. Lee and Ahn^[19]^ evaluated 35 patients and found 4 (11.4%) patients with signs of screw loosening. Gleenie et al^[24]^ evaluated 8 patients, and 5 (62.5%) had signs of screw loosening. In preclinical tests, cortical screws showed some biomechanical advantages that should improve the fixation strength. In 2009, Santoni et al^[6]^ performed a cadaveric study that showed that screws with a cortical trajectory had 30% higher resistance to uni-axial pull-out forces, compared to traditional screw insertion methods. Subsequent biomechanical tests showed that CBTT screws required nearly twice the insertion torque required for TT screws, and CBTT screws had a higher resistance to pulling out than TT screws.^[7,8]^ In practice, screw loosening sometimes occurs. To avoid this, the longest and thickest screws possible are used, and the screw tip is anchored to the disc endplate or lateral wall of the vertebrae to achieve bicortical fixation. Another complication was the screw malpositioning observed in 2 patients. Sakaura et al^[25]^ reported a 2.1% rate of screw malpositioning in a group of 95 patients. Marengo et al^[3]^ reported that 4/418 (0.95%) screws were malpositioned and required repositioning. It is necessary to identify anatomical landmarks for entry points and use intraoperative fluoroscopy or navigation to avoid screw malpositioning. Four reoperations were performed in 3 (7.5%) patients in our group. This is not a small percentage, but in other lumbar fusion techniques, the reoperation rate reaches up to 16% to 19%.^[26,27]^

Recent studies have reported other complications, including superior facet joint violations (1.25%–9.1%); symptomatic adjacent segment disease, deep vein thrombosis or pulmonary embolism (3.8%); hematomas (1.1%–2.4%), and infection (1.3%–2.1%).^[2–4,19,25,28]^ We observed no infections in our series; this result might have been related to the use of a shorter incision than that required with traditional techniques. To date, in our experience, longer screws with bicortical fixation and filling the intervertebral space with bone chips appeared to be very important for achieving long-lasting success. However, further investigations are required to provide evidence in support of these observations.

### 4.4 Limitations of the study

This retrospective study lacked a control group. Therefore, we could not make direct comparisons to results with, for example, minimally invasive spinal-transforaminal lumbar interbody fusion or TT transpedicular screw fixation. In addition, we did not perform radiological evaluations of the preoperative and follow-up sagittal alignment of the spine, because the main indications for this technique were symptoms of intervertebral foraminal stenosis. Therefore, we could not establish any associations between the clinical results and sagittal balance, based on the collected data.

## 5 Conclusion

We found that the CBTT offered high efficacy in the achievement of spinal fusion and displayed a moderate risk of hardware complications. CBTT achieved clinical improvement in over 90% of patients with lumbar degenerative disease, at a mean follow-up of 4.4 years.

## Author contributions

MB completed material and drafted the manuscript. MB, AB, PK, SK completed the analysis. MB and AB assessed radiological analysis. SK assessed statistical analysis. PK and AM supervised the analysis and critically revised the manuscript. All authors provided substantial intellectual contributions and approved the final version of the manuscript.

**Conceptualization:** Mateusz Bielecki, Przemysław Kunert.

**Data curation:** Mateusz Bielecki, Przemysław Kunert.

**Formal analysis:** Mateusz Bielecki, Artur Balasa, Sławomir Kujawski, Przemysław Kunert.

**Investigation:** Mateusz Bielecki, Artur Balasa, Przemysław Kunert.

**Methodology:** Mateusz Bielecki, Przemysław Kunert.

**Resources:** Mateusz Bielecki.

**Supervision:** Przemysław Kunert, Andrzej Marchel.

**Validation:** Mateusz Bielecki, Przemysław Kunert.

**Visualization:** Mateusz Bielecki.

**Writing original draft:** Mateusz Bielecki.

**Writing – review & editing:** Mateusz Bielecki, Przemysław Kunert.
